# Sterile, recurrent, and bilateral corneal perforation related to primary biliary cirrhosis complicated by secondary Sjögren syndrome and vitamin A deficiency

**DOI:** 10.5935/0004-2749.20210100

**Published:** 2025-08-21

**Authors:** Leyre Lloreda Martín, Carlos Rocha-de-Lossada, Sara Marín-Martínez, Jorge Ernesto Peraza-Nieves

**Affiliations:** 1 Department of Ophthalmology, Hospital Universitario Fundación Alcorcón, Madrid, Spain; 2 Department of Ophthalmology, Hospital Clínic de Barcelona, University of Barcelona, Spain

**Keywords:** Primary biliary cirrhosis, Corneal perforation, Sjogren syndrome, Vitamin A deficiency, Autoimmune disease, Cirrose hepática biliar, Perfuração da córnea, Síndrome de Sjogren, Deficiência de vitamina A, Doença autoimune

## Abstract

Primary biliary cirrhosis is a rare progressive autoimmune liver disease that causes chronic cholestasis. Of patients with primary biliary cirrhosis, 75% develop secondary Sjogren syndrome and could develop vitamin A deficiency. Here, we report the case of a patient with primary biliary cirrhosis who developed a secondary Sjogren syndrome and vitamin A deficiency, which led to severe and unusual eye involvement with multiple and recurrent spontaneous corneal perforations. Corneal perforations in patients with primary biliary cirrhosis and secondary Sjogren syndrome are rare but devastating complications, in contrast to other eye clinical manifestations of the disease.

## Abstract (pt)

A cirrose biliar primária é uma doença hepática autoimune progressiva rara que causa colestase crônica. 75% dos pacientes com Cirrose Biliar Primária desenvolvem Síndrome de Sjögren Secundária, e podem também desenvolver deficiência de vitamina A. Aqui, relatamos um paciente com Cirrose Biliar Primária que desenvolveu Síndrome de Sjögren Secundária e deficiência de vitamina A, levando a envolvimento ocular grave e incomum com perfurações espontâneas múltiplas e recorrentes da córnea. Perfurações da córnea em pacientes com Cirrose Biliar Primária e Síndrome de Sjögren Secundária são complicações raras, mas devastadoras, em contraste com outras manifestações clínicas oculares da doença.

## INTRODUCTION

Primary biliary cirrhosis (PBC) is a progressive disease of the liver caused by chronic nonsuppurative destructive cholangitis and destruction of interlobular bile ducts, which results in cholestasis^(1)^. As the disease progresses, cirrhosis and liver failure occur. PBC has four stages. Although the disease is called “primary biliary cirrhosis,” cirrhosis only develops in stage 4, when the damage is permanent and severe scarring of the liver has occurred^(1)^.

Women are nine times more likely than men to develop PBC^(1,2)^, usually at ages 40-60 years. The prevalence is low, estimated between 1 and 5 per 10,000 population, depending on the age and sex^(1)^.

Up to a quarter of patients with PBC are asymptomatic at the time of diagnosis, with the disease found incidentally because of abnormal liver blood test results^(2)^. The most common initial symptoms are fatigue, severe skin itching, hyperpigmentation, jaundice, and hepatomegaly. Complications of PBC include cirrhosis, portal hypertension, osteoporosis, and malabsorption of liposoluble vitamins (vitamins A, D, E, and K). Vitamin A deficiency may produce ocular manifestations such as nyctalopia or xerophthalmia^(1,2)^.

PBC is associated with many other immune manifestations such as and antimitochondrial antibodies (AMA), which occur in >90% of patients. Of patients, 75% develop secondary Sjogren syndrome (SS) with eye involvement^(3)^.

To the best of our knowledge, this is the first report describing a patient with PBC, secondary SS, and vitamin A deficiency who developed progressive and multiple spontaneous corneal perforations.

## CASE REPORT

We present the case of a 45-year-old Moroccan woman with a history of severe PBC, stage IV, which was diagnosed in 2000 on the basis of positive antimitochondrial antibody in serum (titer > 1:40 in enzyme immunoassay) and treated with oral ursodeoxycholic acid since then. Owing to the progression of the disease, she consequently developed portal hypertension, esophageal varices, vitamin A deficiency (10 µg/dl), splenomegaly, and cirrhosis. The patient had no ophthalmologic antecedents, but she developed a secondary severe SS, so therapy with multiple artificial tear preparations was prescribed without improvement. Her tear film breakup time (BUT) was already altered (0-1) when she first visited our hospital.

**Figure 1:**
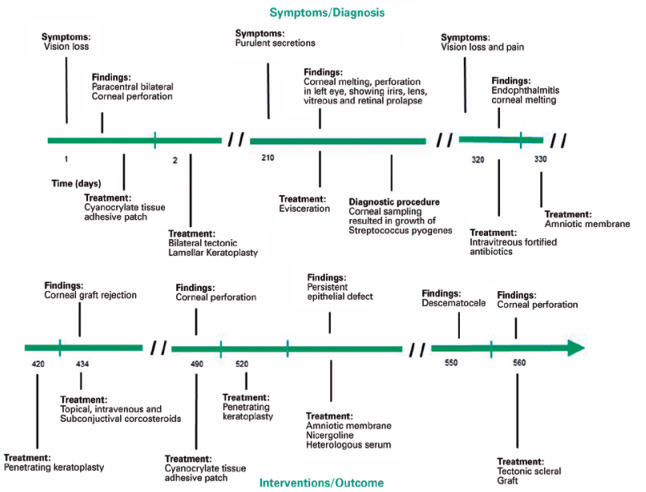
Clinical timeline of the disease.

A statement of consent to publish this case and the images was obtained from the patient. A clinical timeline of the disease is presented in figure 1.

In April 2018, the patient was referred to our center (a *cornea* transplant *reference hospital*) from a regional hospital, so information about the previous status of the patient could not be obtained. During her first visit to our hospital, she presented with painless, paracentral, simultaneous bilateral corneal perforations.

**Figure 2:**
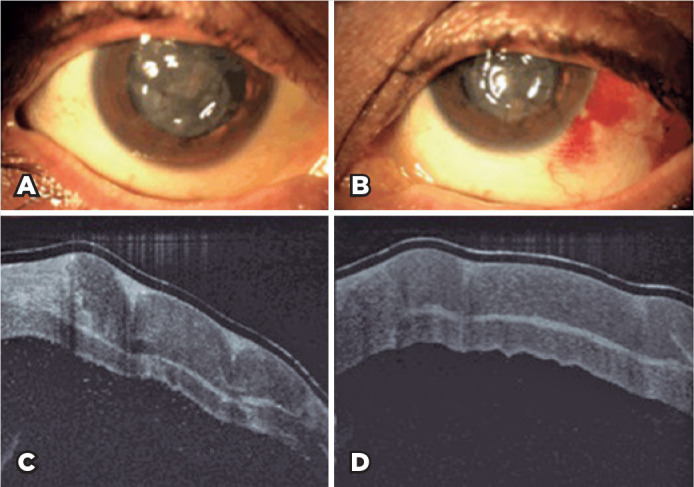
The slit-lamp images show bilateral tectonic lamellar keratoplasty of the A) right eye and B) left eye. The bilateral anterior segment optical coherence tomography images show tectonic corneal grafts of the C) right eye and D) left eye.

A cyanoacrylate tissue adhesive patch was placed on both eyes, which resulted in no leaking aqueous. A week later, bilateral tectonic lamellar keratoplasty was successfully performed (Figure 2). After the keratoplasty, the immediate treatment prescribed included a topical steroid and antibiotic eye drops (TobraDex) 5 times a day, and cycloplegic eye drops (cyclopentolate 1%) 3 times a day.

In November 2018, she was admitted because of corneal melting, abundant purulent secretions, and perforation in her left eye, showing iris, lens, vitreous, and retinal prolapse throughout the perforation; thus, ocular evisceration was performed. During the intervention, a scleral abscess was observed, and no orbital implant was placed in the anophthalmic cavity. A corneal sample obtained during surgery showed growth of *Streptococcus pyogenes*.

In January 2019, the patient was scheduled for a penetrating keratoplasty (PK) associated with amniotic membrane implantation (AMI) in her right eye (OD), but it was suspended owing to alterations of the coagulation parameters resulting from the PBC.

After 2 months, the patient was admitted to the emergency department after referral from another hospital under the diagnosis of endophthalmitis treated with intravitreous fortified antibiotic injections with ceftazidime (2.25 mg/0.1 ml) and vancomycin (1.0 mg/0.1 ml). The best-corrected visual acuity was hand motions, and we observed an epithelial defect of 6.5 × 4.0 mm and corneal melting resulting in 230 microns of minimum graft thickness.

Fortified eye drops of ceftazidime (50 mg/ ml) and vancomycin (50 mg/ml) and systemic moxifloxacin (400 mg/24 hours) were used to control the infection. Ten days later, a double AMI was performed.

In June 2019, a PK associated with AMI was performed. Two weeks after the procedure, the patient presented with corneal graft rejection with an epithelial defect, 360^o^ corneal neovascularization, corneal edema, and keratic precipitates. Topical corticosteroids (1% prednisolone acetate) were prescribed hourly, and a single pulse of intravenous methylprednisolone (500 mg) and subconjunctival triamcinolone (20 mg) were administrated. Three months later, the graft rejection was reversed, with a persistent corneal epithelial defect.

**Figure 3:**
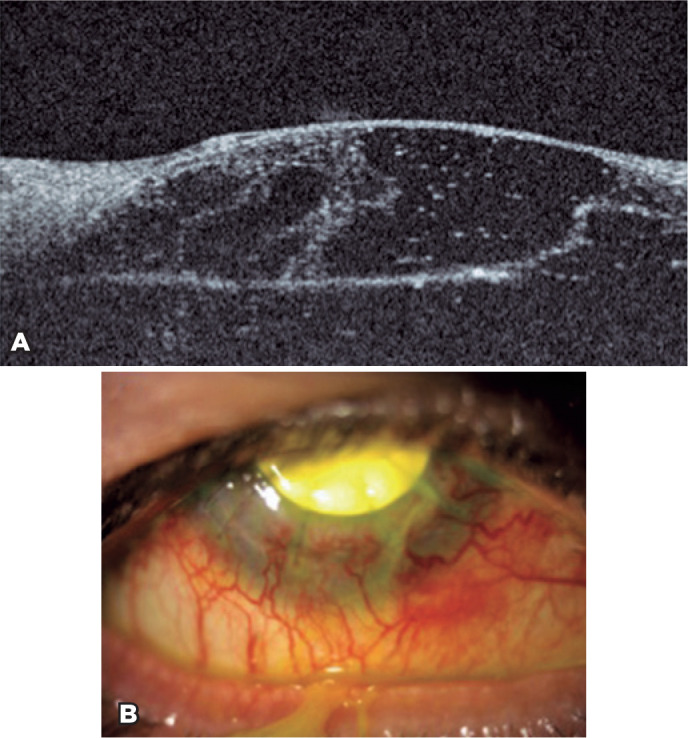
A) Anterior segment optical coherence tomography image showing a descemetocele. B) Tectonic scleral graft.

Another AMI to treat the persistent corneal epithelial defect was performed. The patient was treated with artificial preservative-free tears, heterologous serum eye drops 6 times per day, and 10 mg of oral nicergoline each 12 hours^(4)^. However, the patient had another corneal perforation at 3 months of follow-up, 2 weeks after developing a descemetocele. A tectonic scleral graft was implanted (Figure 3). The evolution of the patient was not as good as expected. In August, she developed endophthalmitis again, with severe corneal melting, and choroidal detachments, with null response to treatment. Unfortunately, she was finally eviscerated.

## DISCUSSION

Herein, we present the case of a female patient with PBC complicated with SS and vitamin A deficiency with 5 corneal perforations and ocular inflammation due to an uncontrolled systemic disease.

PBC is a progressive disease of the liver that causes chronic cholestasis, mainly affecting women. Of patients with PBC, 25% are asymptomatic at the time of diagnosis, but as the disease progresses, complications such as portal hypertension, osteoporosis, and liposoluble vitamin deficiency (vitamins A, D, E, and K) can occur^(1,2)^.

PBC is associated with many other immune manifestations such as SS (75%) with eye involvement^(2,3)^, with AMA being positive in >90% of patients^(3,5)^. Our patient showed high AMA levels and, consequently to her disease, developed a severe secondary SS with multiple ocular perforations. Eye involvement is also related to vitamin A deficiency, resulting from malabsorption of liposoluble vitamins in the context of PBC^(5)^. Vitamin A deficiency affects the goblet cells of the conjunctiva and holocrine glands, which results in mucin deficiency that leads to severe dry eye syndrome and complications such as keratomalacia and perforation^(6)^. Our patient developed vitamin A deficiency secondary to her liver disease. This could be the cause of the delayed corneal epithelial healing and subsequent neurotrophic corneal ulcer, corneal thinning, and multiple spontaneous corneal perforations.

To our knowledge, this is the first report describing a patient with PBC, secondary SS, and vitamin A deficiency who developed progressive and multiple spontaneous uncontrollable corneal perforations.

Corneal thinning and perforation are known to occur in SS. It is often precipitated by topical corticosteroid use along with a systemic condition^(7-10)^. The particularity of our case is that the first spontaneous corneal perforation occurred even in the absence of corticosteroid use; this was probably due to the severe secondary SS concomitant with severe vitamin A deficiency.

In conclusion, PBC is a rare autoimmune liver disease. Although a high percentage of patients developed secondary SS and vitamin A deficiency, PBC occurring with bilateral simultaneous corneal perforation is an unusual presentation. As far as we now, the present case is the first case reported so far. As explained previously, although the literature describes cases involving SS or vitamin A deficiency associated with PBC with spontaneous corneal perforations, all the cases were finally solved successfully with keratoplasty and treatment of the primary disease^(6,8,10)^. This case is exceptional, as it involved bilateral, multiple spontaneous corneal perforations that were unresponsive to any treatment, systemic or topical.

In this case, the clinical treatment failure was due to the severity of the primary disease, which affected several organs of the patient, specifically her eyes.

Given the magnitude and clinical impact of the eye involvement in this disease, ophthalmologic evaluation must be considered in the multidisciplinary approach for patients with PBC.

## References

[1] Purohit T, Cappell MS. (2015). Primary biliary cirrhosis: Pathophysiology, clinical presentation
and therapy. World J Hepatol.

[2] Sun Y, Zhang W, Li B, Zou Z, Selmi C, Gershwin ME. (2015). The coexistence of Sjögren’s syndrome and primary biliary
cirrhosis: a comprehensive review. Clin Rev Allergy Immunol.

[3] Zhu Y, Ma X, Tang X, Hua B. (2016). Liver damage in primary biliary cirrhosis and accompanied by
primary Sjögren’s syndrome: a retrospective pilot
study. Cent Eur J Immunol.

[4] Lee YC, Kim SY. (2015). Treatment of neurotrophic keratopathy with
nicergoline. Cornea.

[5] Whaley K, Goudie RB, Williamson J, Nuki G, Dick WC, Buchanan WW. (1970). Liver disease in Sjögren’s syndrome and rheumatoid
arthritis. Lancet.

[6] Kopecký A, Benda F, Němčanský J. (2018). Xerosis in Patient with Vitamin A deficiency - a case
report. Cesk Slov Oftalmol.

[7] Citirik M, Berker N, Kacar S, Kekilli M. (2012). Ocular findings in patients with autoimmune liver
disease. Ocul Immunol Inflamm.

[8] Alarcón-Segovia D, Díaz-Jouanen E, Fishbein E. (1973). Features of Sjögren’s syndrome in primary biliary
cirrhosis. Ann Intern Med.

[9] Giovannini A, Ballardini G, Amatetti S, Bonazzoli P, Bianchi FB. (1985). Patterns of lacrimal dysfunction in primary biliary
cirrhosis. Br J Ophthalmol.

[10] Krachmer JH, Laibson PR. (1974). Corneal thinning and perforation in Sjögren’s
syndrome. Am J Ophthalmol.

